# Murine Models of Alcohol Consumption: Imperfect but Still Potential Source of Novel Biomarkers and Therapeutic Drug Discovery for Alcoholic Liver Disease

**DOI:** 10.33696/immunology.3.096

**Published:** 2021

**Authors:** Khaled Alharshawi, Costica Aloman

**Affiliations:** Department of Internal Medicine, Division of Digestive Diseases and Nutrition, Rush Medical College, Chicago, IL 60612, USA

**Keywords:** Alcoholic liver disease (ALD), Mouse, Meadows-Cook (MC), Lieber-DeCarli (LD), Patatin-like phospholipase domain containing 3 (Pnpla3), Lipocalin-2 (Lcn2), ELOVL Fatty Acid Elongase 6 (Elovl6), Sulfotransferase family 2A, member 3 (Sult2a3)

## Abstract

Animal models of liver disease are fundamentally important to strengthen our knowledge and understanding of human liver diseases. Murine models of alcohol consumption are utilized to investigate alcoholic liver injury to develop new therapeutic targets. The well accepted and commonly used murine models of chronic alcohol consumption are Meadows-Cook (MC) and Lieber-DeCarli (LD). LD model is based on an isocaloric high-fat liquid diet, but mice under the MC model fed on a regular chow diet with alcohol added to the drinking water. Alcoholic liver disease in real world is frequently diagnosed in patients with obesity and high fat intake, mirroring LD diet. The overlap of the specific effect of ethanol and obesity is difficult to differentiate by clinician and pathologist. In this commentary, we will further discuss our research findings comparing MC and LD as a tool to dissect early alcohol versus increased fat intake detrimental effects on the liver. The critical analysis of these two models could provide evidence to differentiate the specific impact of alcohol on the liver from the combined influence of alcohol and diet. Ultimately, these investigations could uncover potential biomarkers and therapeutic targets for personalized type of alcoholic liver injury.

## Commentary

Improving our knowledge regarding the cellular and molecular mechanisms underlying murine models of alcoholic liver injury should enhance the management and therapies of alcoholic liver disease (ALD) seen in humans [1]. Although none of the animal models available reproduce all main aspects of human ALD, they still provide very useful tools to study and understand the molecular mechanisms of the human counterpart [2]. There are two well accepted and commonly used murine models of chronic alcohol consumption introduced by Meadows-Cook (MC) and Lieber-DeCarli (LD) [2,3]. In the MC model, alcohol is introduced in drinking water, in a final concentration increased gradually to reach 20% in most cases [2,4]. Mice kept in the MC model for up to 16 weeks; however, most studies use 12 weeks as the final readout point [3]. LD was developed to enhance the alcoholic liver injury phenotype in mice [2,5]. The LD model is based on an isocaloric liquid diet with alcohol concentration usually increased to 3.395% for the duration of 25 days to eight weeks, with four weeks as an average duration used [3]. These two murine models of chronic alcohol consumption represent a potential investigative tool to explore the specific effects of alcohol combined with the high lipid liquid diet (LD) versus solid chow (MC) or alcohol regardless of the diet type (Table 1). Our group’s study [3] characterized the immune cellular, transcripts, and histological phenotypes between LD and MC models with encouraging results.

The histological phenotype in LD exposed mice with or without alcohol showed more steatosis than MC mice [2,3]. This is expected considering the liquid high fat diet in LD. This aspect of high lipid diet with alcohol (LD) which enhanced the hepatic steatosis, could represent the phenotype of alcoholic liver injury in obese people. Considering the obesity problem, at least in western societies, comparing LD and MC will help understand the molecular mechanisms of liver injury induced by alcohol alone and alcohol combined with obesity, i.e., a high fat diet.

Surprisingly, hepatic leukocytes (CD45+) are significantly higher in MC compared to LD in both alcohol-fed and control mice [3]. Further, the majority of the hepatic leukocytes were from lymphoid lineage [3], contrasting with the overall enrichment in innate immune cells of pathological features of these conditions in human. There is evidence that chronic alcohol consumption decreases T cells and B cells in both humans and animal models [8].

Neutrophils and monocytes are critical components of ALD. Although their role is not yet clearly understood, neutrophils are important immune cells affected by alcohol, and their hepatic numbers are suggested to correlate with survival in alcoholic hepatitis [9, 10]. MC and LD models are murine models of early alcoholic liver injury and not models of alcoholic hepatitis; in these very early stages, we did not reveal significant changes in the numbers of hepatic neutrophils between alcohol-fed and control mice in both mouse models [3]. Surprisingly, however, in both alcohol and control, the hepatic neutrophil numbers in LD mice are decreased compared to MC [3]. Monocytes, the other counterpart innate immune cells, play an important role in ALD and potentially a cellular target for new therapeutics [11–13]. Like neutrophils, there was no difference between control and alcohol exposed mice, but mice on the MC model have higher numbers of hepatic monocytes compared to LD [3].

These data indicate that in the LC model, cellular immunological are somehow dimmed by high fat liquid diet and suggest that at least from a cellular immunological perspective, MC model may be potentially a closer representation of human alcoholic liver injury than the LD model. Benefiting from this, our recent study using the MC model to characterize hepatic dendritic cells (DC) revealed a gender dichotomy effect of alcohol response on hepatic plasmacytoid DCs (pDC) [14]. Further, Using the MC diet for four weeks, interestingly, we recently showed an earlier increase in hepatic monocytes only in alcohol-fed female mice compared to their control counterparts [15].

Furthermore, studying transcriptomics in both models unexpectedly revealed only a limited number of genes affected specifically by alcohol, diet type, or the combination [3].

Patatin-like phospholipase domain containing 3 (Pnpla3), a gene coding for a member of lipid hydrolase enzyme [16]. Variants in pnpla3 gene are well known as genetic risk factors for both ALD and non-alcoholic fatty liver disease (NAFLD) [17,18]. In our study by Vogle et al. [3], we found that pnpla3 is the only gene that is upregulated in chronic alcohol exposed mice compared to their counterpart controls in both mouse models [3]. However, in the absence of alcohol exposure LD diet has a different effect: pnpla3 was down regulated in LD control mice compared to MC controls [3]. Significant for our observation in mice, in humans counterpart, suppressive mutation of pnpla3 tested *in vitro* and *in vivo* indicated a beneficial effect on NAFLD and therefore may represent a new therapeutic target for alcoholic induced steatosis [19–21].

Non-alcoholic steatohepatitis (NASH) overlap with alcoholic steatohepatitis (ASH) is difficult to differentiate in clinical practice due to the high prevalence of obesity in the general population and absence of specific pathological changes for these conditions [3,22]. Interestingly, studying differentially expressed genes in these two models hinted at potential biomarkers, which might help differentiate between NASH, ASH, and NASH-ASH combination [3].

Lipocalin-2 (Lcn2), also known as neutrophil gelatinase-associated lipocalin, is expressed by tissues and immune cells in response to inflammation [23–25]. Lcn2 has been investigated in alcoholic liver injury models as well as human alcoholic hepatitis. In human alcoholic hepatitis with advanced fibrosis, Chen et al. investigated the intrahepatic Lcn2 expression and serum levels of Lcn2 [26]. Their study showed, in alcoholic hepatitis patients, a correlation between the disease severity and portal hypertension with increased hepatic Lcn2 expression and Lcn2 serum levels [26]. In LD mouse model, Lcn2 exacerbated the development of ALD, and Lcn2 knockout protected mice from ALD and liver fibrosis [26–28]. In our comparison, Lcn2 expression was found downregulated in MC alcohol-fed mice compared to control and LD alcohol-fed mice [3]. This differential regulation of Lcn2 pointing to a potential use to differentiate ASH, represented in the study by MC alcohol consumed, and NASH-ASH combination, represent by LD alcohol-fed. Lcn2 was found to be a biomarker for the coexistence of liver injury not only induced by alcohol but when there is liver involvement in inflammatory arthritis [29] or in type-2 diabetes mellitus patients with hepatitis B co-infection [30].

Only a few transcripts seem to be affected by alcohol consumption in a similar way in both models and potential common target for therapies for both variants of alcoholic liver injury. ELOVL Fatty Acid Elongase 6 (Elovl6) gene codes an enzyme involved in elongating 16 carbon saturated and unsaturated fatty acids yielding 18 carbon fatty acids [31,32]. Sulfotransferase family 2A, member 3 (Sult2a3) gene express the enzymes involved in catalyzing the sulfate conjugation for many hormones and neurotransmitters [33]. Elov6 and Sult2a3 were upregulated by alcohol consumption in MC and LD compared to controls [3]. This upregulation, regardless of the different diets between the two mouse models, suggests the potential of using them as alcohol-specific therapeutic targets. There are no studies published so far regarding their involvement in alcoholic liver disease pathogenesis. The four genes and their potential biomarker/therapeutic utilization is described in Table 2.

Another layer of complexity is brought by the sexbased differential gene expression on somatic tissues, liver specifically [34]. Pnpla3 variant was found to be a risk factor for reduced survival of males with primary sclerosing cholangitis [35]. Lcn2 has shown a sex specific difference in hepatic steatosis in a mice study [36]. In a diversity outbred mice study, Sult2a3 was assigned to female liver co-regulated cluster [34]. We do not know at present if Elvol6 is the subject of similar modulation.

In summary, our study strongly suggests that at least in the early stages, alcohol effect on the murine liver are mechanistically quite different and model specific. Whether with increasing time of alcohol exposure, this mechanistic difference converges or remains quite distinct, highlights the importance of continuing these studies. These studies suggesting diversity of ALD needs be validated in human as well. The results of such studies might surprise us in a way that alcoholic hepatitis in a slim person may not be the same disease from a mechanistic perspective as in an obese person, and therefore, they should not be treated the same, in spite of their similar histological characteristics.

## Figures and Tables

**Table 1:** Summary description of Meadows-Cook (MC) and Lieber-DeCarli (LD).

Feature	MC	LD	References
Goal of the model	The simplest model developed to investigate the effect of chronic alcohol exposure on the immune system	Developed to enhance the liver injury phenotype to study the initial stages of ALD	[2,3,5–7]
Model concept	Regular chow diet with alcohol added to drinking water	High fat liquid diet with added alcohol	
Duration average	12 weeks	4 weeks	

**Table 2:** Brief description of potential biomarkers genes.

Gene	Literature	Our Finding	Biomarker/ Therapeutic target
Pnpla3	Variants of it are well known genetic risk factors for both ALD and NAFLD [17,18]	The only gene that is upregulated after chronic alcohol exposure in both mouse models and downregulated in LD controls compared to MC controls [3]	Potential common therapeutic target for alcoholic induced steatosis [19–21]
Lcn2	It is associated with increase in liver injury severity in mouse and human [26–28]	Downregulated in MC alcohol-fed mice compared to MC control and LD alcohol-fed mice [3]	Potential use to differentiate ASH from NASH-ASH combination [3,29,30]
Elovl6 & Sult2a3	No studies regarding their involvement in ALD pathogenesis [3]	Upregulated by alcohol consumption in both MC and LD compared to controls [3]	Potential use as target and markers of alcohol-specific immunopathology [3]
